# A continuous analog of run length distributions reflecting accumulated fractionation events

**DOI:** 10.1186/s12859-016-1265-5

**Published:** 2016-11-11

**Authors:** Zhe Yu, David Sankoff

**Affiliations:** Department of Mathematics and Statistics, University of Ottawa, 585 King Edward Avenue, Ottawa, Ontario, K1N 6N5 Canada

**Keywords:** Genomics, Whole genome duplication, Analysis of runs, Probability modeling, Duplicate gene deletion

## Abstract

**Background:**

We propose a new, continuous model of the fractionation process (duplicate gene deletion after polyploidization) on the real line. The aim is to infer how much DNA is deleted at a time, based on segment lengths for alternating deleted (invisible) and undeleted (visible) regions.

**Results:**

After deriving a number of analytical results for “one-sided” fractionation, we undertake a series of simulations that help us identify the distribution of segment lengths as a gamma with shape and rate parameters evolving over time. This leads to an inference procedure based on observed length distributions for visible and invisible segments.

**Conclusions:**

We suggest extensions of this mathematical and simulation work to biologically realistic discrete models, including two-sided fractionation.

## Background

In the course of evolution, new genomes occasionally arise by duplication or triplication of an existing genome, so that there are two or three identical copies of each maternal and each paternal chromosome. After a (usually) transient period of polyploidy marked by unusual patterns of meiosis where more than just one maternal and paternal chromosome are aligned and recombine, processes of sequence divergence and chromosome rearrangement lead to more familiar diploid patterns. At the same time a process of *fractionation* eliminates some or most of the duplicate genes, some from each chromosomal copy, but in the simplest model, never all members of a duplicate pair or triple - for reasons of viability. Fractionation processes have been surveyed across evolutionarily diverse types of eukaryote organisms [1].

Since one copy of a duplicate pair of genes must be retained, we can identify not only the chromosomal regions that have been retained – by simple observation of the genome – but also each region that is now invisible – by reference to the duplicate chromosome that has necessarily retained a copy of this region. Thus, the data on which inferences about the deletion process can be made consist of alternating segments of deleted and undeleted genome of varying lengths.

Among the important questions about the nature of the deletion process, we can ask whether deletion proceeds one gene at a time or by larger chromosomal fragments. In this paper, we model the process as the deletion of segments from the real line, with a biologically realistic treatment afforded to overlapping deletions. Previous work focused on the difficult question of how many overlapping deletion events are responsible for each contiguous deleted region [2–4], but was not able to account analytically for the dynamics of the process.

In the present paper we attack and solve the inference problem of the size, form and spacing of deletion events, allowing for a number of sweeps over the genome as a way of accounting for overlapping deletions. We carry this out in a continuous analog of the original discrete gene-order context, and address the “one-sided” version of the problem, where all deletions occur on one of the duplicate chromosomes.

There has been a certain amount of work on the quantification of the fractionation process, starting in 2006 with [5], which claimed deletions involved one gene at a time, and [6], which treated the number of genes deleted in a single event as a random variable with mean greater than 1. Other work of this kind includes [1] and [7]. However, the modelling of fractionation where the whole genome evolves as a stochastic process began with [2]. The previously unstudied phenomenon taken into account in that work was the overlap of deletion events, something that assumes much importance soon after the fractionation process commences. Overlap must be handled differently if all deletions occur from one copy of the genome or in either copy. To isolate the most important aspect of overlap, [2] gave analytical results for the case where deletions all occurred on one copy (“one-sided” model). Then [3] extended this to the more realistic case where deletion could occur at different rates, or the same rate, from either copy of the genome (“two-sided” model). This analysis was more difficult and could not be taken as far as with the one-sided model.

For the one-sided model, a closed form solution of how many deletion events contribute to a deleted region after a single event (i.e., at a single step in the fractionation process) was obtained in [4].

## Methods

### The proposed model

We model the fractionation process in terms of a number of successive sweeps of a point process with parameter *ν* on the positive reals, i.e., *ν*∈**R** ^+^, representing one copy of the genome. At the origin, we say that all points of this genome are “visible”. A deletion event, rendering a segment of exponentially (mean *μ*) distributed length “invisible”, occurs at each point determined by the point process. The second copy of the genome remains undisturbed throughout and retains a 1-to-1, length preserving, correspondence with the fractionating copy, without regard to any disruption caused by invisibility. In applications, the acceptance of the one-gene-at-a time theory of deletion depends on whether *μ* is below or above a certain absolute value, but the present work is part of the mathematical preliminaries to the practical questions. The eventual goal of this work is to determine the relative size of the “spacing” parameter *ν* and the deletion length parameter *μ*. The model innovation here is to introduce the parameter *ν* in the place of a rate parameter in previous work, which was awkward to work with.

**Fig. 1:**
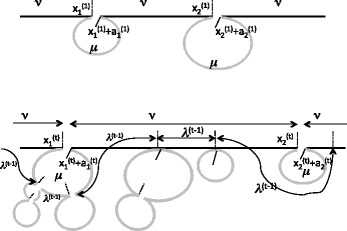
Processes pertinent to first sweep and *t*-th sweep. *Solid horizontal bars* represent the visible regions of the genome. *Grey curves* represent invisible regions. *Dashed markers* represent deletion points, *solid markers* represent end of deletion segments. *ν* and *μ* are the means of the deletion point spacing and deletion segment length variables, while *λ* ^(*t*−1)^ is the mean space (= *λ*_*t*−1_ in the text) between visible deletion points after the *t*−1-st sweep

During the first sweep, illustrated at the top of Fig. 1 at time (or step) *t*=1, the first *deletion point* *x*_1_ is determined by sampling from the exponential distribution

1 $$ \rho(x)=\frac{1}{\nu}e^{-\frac{x}{\nu}},~ x\geq 0,  $$

with mean *ν*. Then a deletion length *a*_1_ is chosen from another exponential distribution

2 $$ \gamma(a)=\frac{1}{\mu}e^{-\frac{a}{\mu}},~ a\geq 0,  $$

with mean *μ*. Normally, *ν*≫*μ*, but this is not necessary to the analysis. The segment [*x*_1_,*x*_1_+*a*_1_) is “deleted", or is designated as invisible. The next deletion point *x*_2_ is chosen by sampling *x*2′ from the first exponential distribution (mean *ν*), so that *x*_2_=*x*2′+*x*_1_+*a*_1_. Then the length *a*_2_ of the second deleted segment is determined by sampling from *γ* again. The process continues in this way to find *x*_3_,*a*_3_,… Concatenating only those segments that are still visible, we see that *x*_1_,*x*_2_,… are points determined by a point process with parameter *ν*. Associated with each of these points *x* is an “event counter" *C*(*x*). Initially, each *C*(*x*)=1. We define a function *π*_*t*_(*i*),*i*=1,… measuring the proportion of event counters registering *i* events at time *t*≥1. Thus *π*_1_(1)=1 and *π*_1_(*j*)=0, for all *j*>1.

At times *t*=1,2,…, the second, third, … sweeps begin, all independent of the first sweep and each other, and each applied to the concatenated visible segments only. We sample $x_{1}^{(t)}$ and $a_{1}^{(t)}$ in the same way as *x*_1_ and *a*_1_ according to *ρ* and *γ*, respectively, to determine a deletion interval $[x_{1}^{(t)}, x_{1}^{(t)}+a_{1}^{(t)})$.

If the interval $[x_{1}^{(t)}, x_{1}^{(t)}+a_{1}^{(t)})$ contains no previously defined deletion point, a new event counter at $C(x_{1}^{(t)})$ is set at 1. If $[x_{1}^{(t)}, x_{1}^{(t)}+a_{1}^{(t)})$ already contains *j*>1 deletion points *z*_1_,…,*z*_*j*_, the event counter at $C(x_{1}^{(t)})$ is set at $1+\sum _{i=1}^{j} C(z_{i})$. The *j* deletion points *z*_1_,…,*z*_*j*_ become invisible, along with the rest of the segment $[x_{1}^{(t)}, x_{1}^{(t)}+a_{1}^{(t)})$ that contains them.

We find the next deletion point by sampling $x_{2}^{(t)'}$ from *ρ*, and setting $x_{2}{(t)}=x_{1}^{(t)}+a_{1}^{(t)}+x_{2}^{(t)'}$. We continue the *t* sweep, adding visible deletion points and making others invisible. Some deletion points from the earlier sweep will remain unchanged, i.e. are still visible. The $x_{i}^{(t)}$ by themselves define a point process with parameter *ν* on the concatenated visible segments. But the $x_{i}^{(t)}$ and the additional deletion points remaining from the earlier sweep define a process with mean *λ*_*t*_, a parameter that decreases with *t*, as the undeleted segments are interrupted by more and more deletions. This parameter is important as it is directly inferable from the observed genome at time *t*.

More important, it is clear, that at each sweep, more and more of the genome becomes invisible. Since each concatenation of visible segments still extends to the positive reals, we cannot observe directly how much the genome has been reduced in absolute terms. But thanks to the length-preserving isomorphism between the second copy of the genome and the fractionating one, for any large finite interval we can observe the proportion of the genome that is left by time *t* and we can predict that it is approximately $(1-\frac {\mu }{\nu +\mu })^{t}$.

We will calculate *λ*, the number of deletion points in [*x*_*i*_,*x*_*i*+1_), the distribution *p*(*j*),*j*=1,... of the number *j* of pre-existing deletion points in intervals deleted during each sweep, and discuss how to calculate *π*_1_(*j*),*j*≥1, the proportion of event counters with *C*=*j*.

## Results

### The length of undeleted segments *λ*

After the first sweep, *x*_*i*_ is the only deletion point in $[x_{i}^{(1)},a_{i}^{(1)})$ and the only deletion point in the visible $[x_{i}^{(1)}, x_{i+1}^{(1)})$, so that *λ*_1_=*ν*. During the second sweep, the number of these first-sweep deletion points that the visible $[x_{i}^{(2)},x_{i+1}^{(2)})$ contains is Poisson distributed with mean $\frac {\nu }{\nu +\mu }$, while the remaining first-sweep deletion points that the invisible $[x_{i}^{(2)},a_{i}^{(2)})$ contains are Poisson distributed with mean $\frac {\mu }{\nu +\mu }$. (These are approximations, since the true means are $\frac {x_{i+1}^{(2)}-x_{i}^{(2)}}{x_{i+1}^{(2)}+a_{i}^{(2)}-2x_{i}^{(2)}}$ and $\frac {a_{i}^{(2)}-x_{i}^{(2)}}{x_{i+1}^{(2)}+a_{i}^{(2)}-2x_{i}^{(2)}}$, respectively.) In addition the visible segment contains one new deletion point, created during the second sweep itself. We can then predict *λ*_2_ to be roughly

3 $$ \hat{\lambda}_{2}=\frac{\nu}{1+\frac{\nu}{\nu+\mu}}.  $$

Suppose *λ*_*t*−1_ is the parameter of the point process that generates the deletion points visible after sweep *t*−1. Then, in the sweep at time *t*, the number of deletion points that the invisible $[x_{i}^{(2)},a_{i}^{(2)})$ will contain is Poisson distributed with mean $\frac {\mu }{\lambda _{t-1}}$. The number of deletion points in the visible [*x*_*i*_,*x*_*i*+1_), not including *x*_*i*_, is Poisson distributed with mean $\frac {\nu }{\lambda _{t-1}}$. In addition, the visible segment contains one new deletion point, created during the *t*-th sweep itself. *λ*_*t*_ can thus be predicted to be approximately

4 $$ \hat{\lambda}_{t}=\frac{\nu}{1+\frac{\nu}{\hat\lambda_{t-1}}}.  $$

Since $\hat {\lambda }_{1}=\nu $,

5 $$ \hat{\lambda}_{t}=\frac{\nu}{t}.  $$

### The treatment of overlapping deletions

The discussions in this section and the next do not depend on *t*, so let *Λ* be the exponential distribution with mean *λ*. From [4], the probability *p*_0_ that a deletion event contains no extant deletion points is

6 $$ p_{0} =\int_{l=0}^{\infty} \frac{l\Lambda(l)}{\lambda}\int_{x=0}^{l}\frac{1}{l}\int_{y=0}^{l-x}\gamma(y)dy\,dx\,dl.  $$

Carrying out the integrations, we find

7 $$ p_{0}=\frac{\lambda}{\mu+\lambda}.  $$

The probability *p*_1_ that a deletion event overlaps exactly one existing run of deletions is:

8 $$\begin{array}{@{}rcl@{}} p_{1}&=&\frac{1}{\lambda}\int_{l=0}^{\infty}\int_{z=0}^{\infty} \Lambda(l)\Lambda(z)\int_{x=0}^{l}\int_{y=l-x}^{l-x+z}\gamma(y)dy\, dx\, dz\, dl\\ && \end{array} $$

9 $$\begin{array}{@{}rcl@{}} &=&\frac{\lambda}{\mu+\lambda}\cdot\frac{\mu}{\mu+\lambda}. \end{array} $$

It can be proved by induction that the probability a deletion event overlaps exactly *q* existing runs of deletions is:

10 $$ p_{q}=\frac{\lambda}{\mu+\lambda}\left(\frac{\mu}{\mu+\lambda}\right)^{q}.  $$

Thus we have the surprisingly uncomplicated result that the number *q* of pre-existing runs of single-copy regions overlapped by a new deletion event is geometrically distributed on *q*=0,1,… with parameter *μ*/(*μ*+*λ*).

### The distribution of event counts *π*

The event count *C*(*x*) at a visible deletion point *x* tells us how many deletion events have occurred to make up the invisible segment adjacent to *x*. In contrast to the undeleted segments, where we know that no events occurred, observing that a segment has been deleted does not tell us *C*(*x*). Some work has focused on the distribution *π*(*i*) of the probabilities that a deletion point *x* has *C*(*x*)=*i*, and we are able to calculate how *π* changes with each sweep. Then we can update *π*_*t*_ by a linear combination of the distribution of changes due to the deletion and the existing *π*_*t*−1_. Let *Δ*(*i*) represent the change in *π*_*i*_ at any sweep *t*. This can be calculated from Eq. (10) and the net effect that a deletion overlapping *q* existing runs has on the various *π*. Without giving details here,

11 $$\begin{array}{*{20}l} \Delta(1)&= p_{0} - p_{1} \left[ \pi(1) \right] - 2p_{2} \left[ \pi(1) \right] - 3p_{3} \left[ \pi(1) \right] \\ & \quad - 4p_{4} \left[ \pi(1) \right] - \ldots \end{array} $$

12 $$\begin{array}{*{20}l} \Delta(2) &\!= p_{1} \left[ \pi(1) \right] - p_{1} \left[ \pi(2) \right] - 2p_{2} \left[ \pi(2) \right] -\! 3p_{3} \left[ \pi(2) \right] \\ &\quad - 4p_{4} \left[ \pi(2) \right] -... \end{array} $$

13 $$\begin{array}{*{20}l} \Delta(3) &= p_{1} \left[ \pi(2) \right] + p_{2} \left[ \left(\pi(1) \right)^{2} \right] - p_{1} \left[ \pi(3) \right] \\ &\quad- 2p_{2} \left[ \pi(3) \right] - 3p_{3} \left[ \pi(3) \right] - 4p_{4} \left[ \pi(3) \right] -... \end{array} $$

14 $$\begin{array}{*{20}l} \Delta(4) &= p_{1} \left[ \pi(3) \right] + 2p_{2} \left[ \pi(1) \pi(2) \right] + p_{3} \left[ \left(\pi(1) \right)^{3}\right]\\ &\quad - p_{1} \left[ \pi(4) \right] - 2p_{2} \left[ \pi(4) \right] - 3p_{3} \left[ \pi(4) \right]\\ &\quad- 4p_{4} \left[ \pi(4) \right] - \ldots  \end{array} $$

15 $$\begin{array}{*{20}l} \Delta(5) &= p_{1} \left[ \pi(4) \right] + p_{2} \left[ 2\pi(1) \pi(3) + \left(\pi(2) \right)^{2} \right] \\ &\quad + 3p_{3} \left[ \left(\pi(1) \right)^{2} \pi(2) \right] + p_{4} \left[ \left(\pi(1) \right)^{4} \right]\\ &\quad - p_{1} \left[ \pi(5) \right] - 2p_{2} \left[ \pi(5) \right] - 3p_{3} \left[ \pi(5) \right] \end{array} $$

16 $$\begin{array}{*{20}l} &\quad- 4p_{4} \left[ \pi(5) \right] -5p_{5} \left[ \pi(5) \right] - \ldots \\ \ldots \end{array} $$

Unfortunately, even knowing the dynamics of *C* does not help us with the inference problem, since the number of events associated with an invisible segment, is not directly associated with the total length of the segment. It is known that the overlapping gamma variables making up each segment are related in a complex way, and cannot simply be treated as the sum of gammas drawn a single population.

This leads us to the approach in the next two sections, where simulations strongly suggest the functional form of the distribution of invisible segment lengths, including shape and rate parameters that can be observed, leading to inference of the simulation parameters based on the observations.

### Simulation

Our simulation experiments were based on initial visible segments of length 10,000, which is very long in comparison to the deletion lengths with *μ*≤10. In other words, we do not risk artificial effects, like a disappearing genome, after a few sweeps, *t*≤10. Moreover, after each sweep, if the total undeleted length =*L*, we add, to the end of the remaining visible portion, segments where the lengths of the visible portions total 10,000−*L*, copied from a replicate trial. The program, written in Java, was repeated 5 times for each configuration of the parameters *μ*,*ν* and *t*. Each set of 5 trials averaged a total of less than 3 min on a Lenovo Y50 laptop.

After each sweep, we calculated the distribution of segment lengths for both the invisible and visible parts of the model genome.

### Parameter estimation

**Fig. 2:**
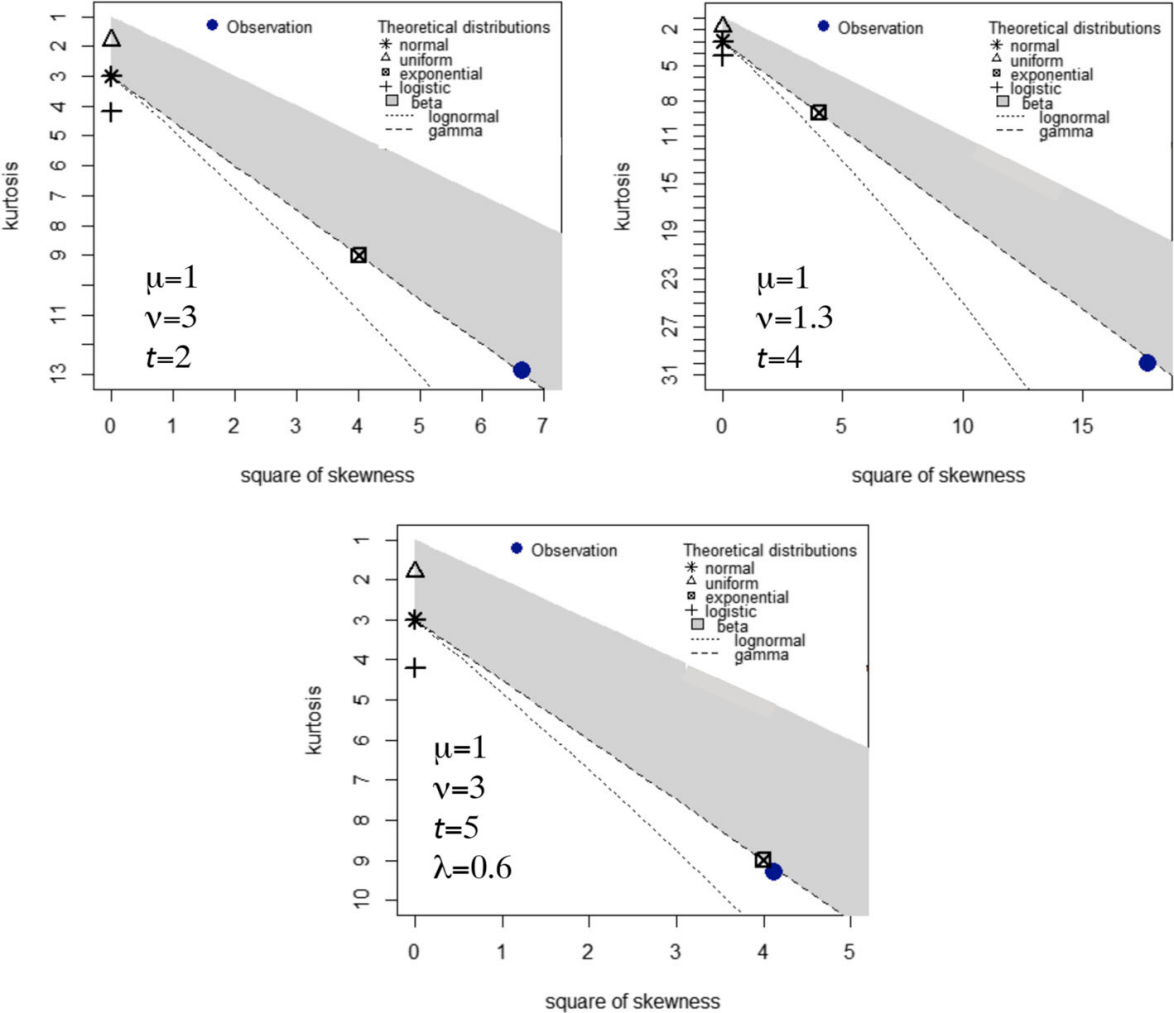
Cullen-Frey diagrams for length distributions of invisible (*top*) and visible (*bottom*) segments

The results of the simulations strongly suggest that the lengths of the invisible segments are gamma distributed, as illustrated in the Cullen-Frey graphs at the top of Fig. 2. As the parameters *ν*,*μ* and *t* change, the moments of the simulated distributions also change, but remain those of a gamma distribution. Similarly, the distribution of the lengths of the visible segments is always exponential, as at the bottom of Fig. 2, with rate

17 $$ \lambda^{-1}=\frac{t}{\nu}.  $$

As a first step towards the ability to infer *μ* and *ν* from the length distributions of invisible and visible segments, we would like to predict *α* and *β*, the shape and rate parameters of the gamma distribution, from *t*,*μ* and *ν*. Table 1 suggests, for a fixed value of *t* and a fixed value $\frac {\mu }{\nu }$, that shape is constant as *μ* changes, and that the rate is inversely proportion to *μ*.

**Table 1:** Simulated values of shape and rate when $\frac {\mu }{\nu }=\frac {1}{3}$, for a range of values of *μ*, and *t*=2

*μ*	*ν*	shape *α*	rate *β*	1/*β*
1	3	0.8994863	0.7801536	1.281798866
2	6	0.8713054	0.3645944	2.742773888
3	9	0.8943245	0.2557653	3.909834524
4	12	0.8551860	0.1863732	5.365578313
5	15	0.8479933	0.1504409	6.647128540
6	18	0.8673458	0.1250687	7.995605615
7	21	0.8793444	0.1044622	9.572840702
8	24	0.91907486	0.09607099	10.40896945
9	27	0.91503151	0.08817842	11.34064321
10	30	0.82931206	0.07483308	13.36307419

**Fig. 3:**
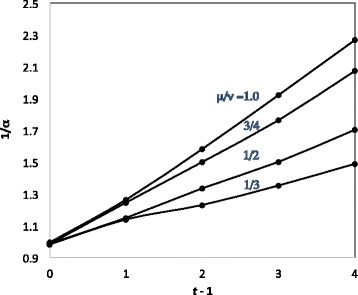
Linear relation between 1/*α* and *t*−1 for fixed $\frac {\mu }{\nu }$

Similar results hold for each combination of *t* and $\frac {\mu }{\nu }$, with different shape constants and rate proportions. Figure 3 shows how the shape constant varies with *t* for four values of $\frac {\mu }{\nu }$.

**Fig. 4:**
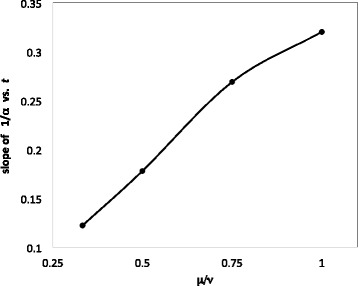
Relation between slope of 1/*α* as a function of *t*, and $\frac {\mu }{\nu }$

The four coefficients of the linear relationships inferred from Fig. 3 are plotted in Fig. 4. Fitting this curve with a quadratic yields

18 $$ \alpha^{-1}-1 =[-0.1725(\frac{\mu}{\nu})^{2}+0.5333\frac{\mu}{\nu}-0.039](t-1).  $$

**Fig. 5:**
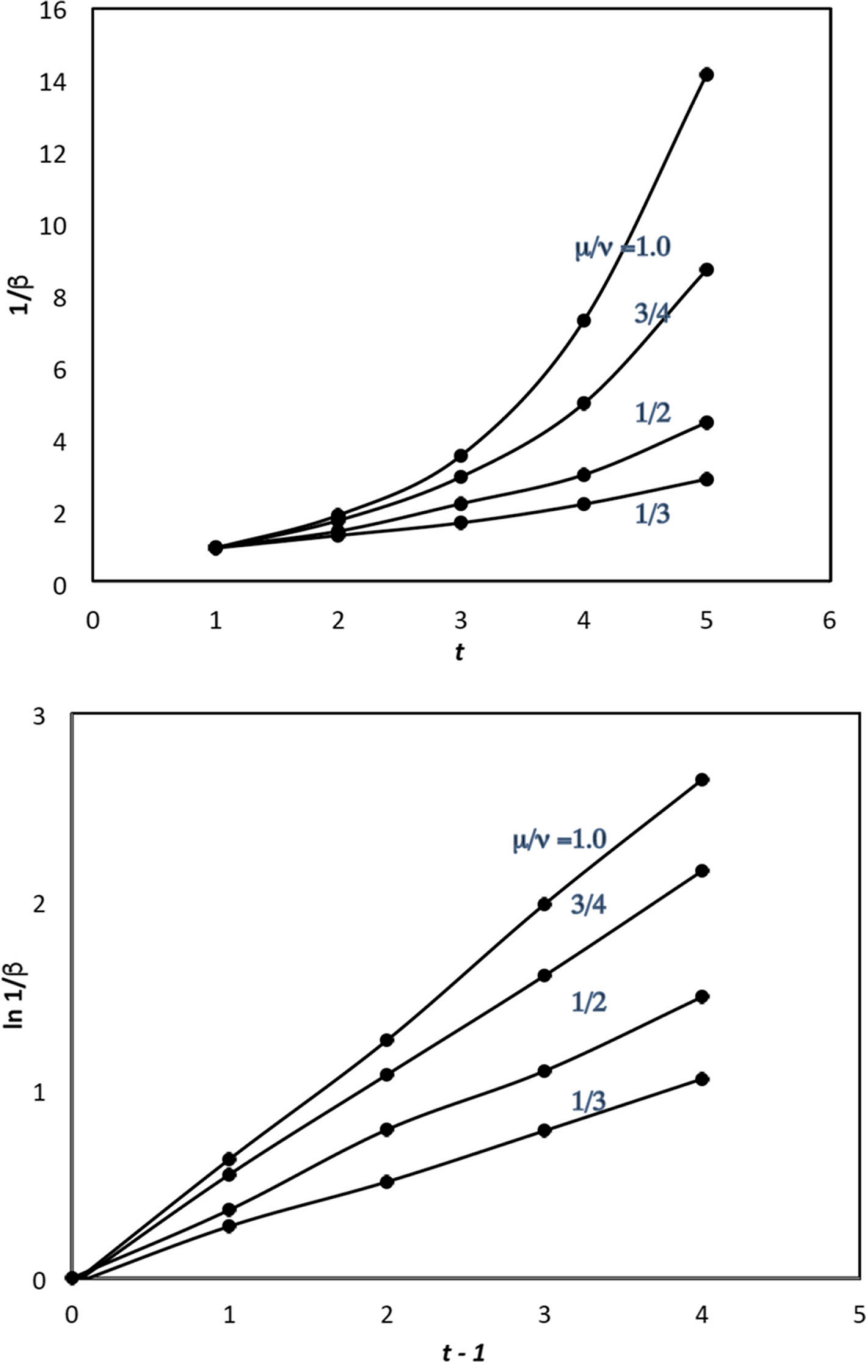
Relation between 1/rate (1/*β*) as a function of *t* for fixed $\frac {\mu }{\nu }$

As for the rate parameter of the gamma, Fig. 5 shows that it is the logarithm of the rate that behaves linearly over time for a fixed value of $\frac {\mu }{\nu }$.

**Fig. 6:**
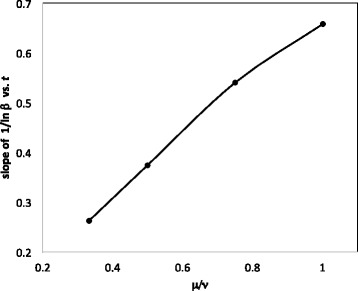
Relation between slope of ln1/*β* as a function of *t*, and $\frac {\mu }{\nu }$

The four coefficients of the linear relationships inferred from Fig. 5 are plotted in Fig. 6. Fitting this curve with a quadratic yields

19 $${} \beta^{-1}=\mu\exp\left[\!\!\left(\!-0.2458\left(\!\frac{\mu}{\nu}\!\right)^{2}\!+0.9257\frac{\mu}{\nu}\!-0.0212\!\right)\!\!(t-1)\!\!\right]  $$

The observable quantities in our model are the distribution of visible segment lengths, predicted to be exponential with mean *λ*, and the shape and rate parameters *α* and *β* of the predicted gamma distribution of invisible segment lengths. These three observable quantities are related to the unknown model parameters *μ*,*ν*, and *t* through Eqs. (17), (18) and (19). With the given value of these parameters, we can estimate the values of *μ*,*ν*, and *t*.

Lacking a closed form solution for *μ*,*ν*, and *t* in terms of *λ*,*α* and *β*, we use the following procedure. Since *t* must be an integer, we can find values of *ν*_*t*_ and *μ*_*t*_ for each *t*=1,2,… with Eqs. (17) and (18). Then we can solve Eq. (19) to find *β*_*t*_.

We then compare all the *β*_*t*_, for *t*=1,2,… with the *β* observed in the simulation, and set

20 $$ \hat{t}= \arg\min{\left\{\frac{\beta-\beta_{t}}{\beta}\right\}}  $$

As an example, in one set of simulations where *μ*=1,*ν*=3 and *t*=5, the experimental value of parameters are *λ* ^−1^=1.665595,*s* *h* *a* *p* *e*=0.6711252 and *β*=0.3504422. When *t*≤2, there is no solution for *μ*. For *t*>2, Table 2 shows the results of this procedure, where 100*δ* is 100 × the normalized difference between *β* and *β*_*t*_ in Eq. (20).

**Table 2:** *μ*=1,*ν*=3,*t*=5,*λ* ^−1^=0.16656,*α*=0.6711,*β*=0.3504

par ∖time *t*	3	4	5	6	7
*μ*	1.231661635	1.063543459	1.021578166	1.018615845	1.033112259
*ν*	1.801158145	2.401544193	3.001930241	3.602316289	4.202702338
*β*_*t*_	0.30056014	0.338494894	0.338654644	0.325314125	0.306788889
100*δ*	14.23403354	3.409208693	**3.363623543**	7.170390806	12.4566366

Bold entry indicates the *t* most consistent with the observed data on *α*,*β* and *λ*

The minimum value of 100*δ* occurs when *t*=5, expressing the fact that the inferred values of *μ* and *ν*, together with *t*=5, are the parameter values most consistent with the observed values of *α*,*β* and *λ*. Other typical examples spanning a range of parameter values are given in Tables 3, 4 and 5.

**Table 3:** *μ* = 6,*ν* = 12,*t* = 2,*λ* ^−1^=0.17,*α*=0.8488,*β*=0.12063

par ∖time *t*	2	3	4	5	6	7
*μ*	5.7892	4.7235	4.7286	4.9648	5.2990	5.6560
*ν*	12	18	24	30	36	42
*β*_*t*_	0.1195	0.1406	0.1342	0.1220	0.1094	0.0976
100*δ*	**0.9107**	16.5215	11.2325	1.1655	9.3410	19.0791

Bold entry indicates the *t* most consistent with the observed data on *α*,*β* and *λ*

**Table 4:** *μ*=1,*ν*=3,*t*=3,*λ* ^−1^=1.017737,*α*=0.7977859,*β*=0.5649623

par ∖time *t*	2	3	4	5	6
*μ*	1.4006	1.0332	0.9909	1.0102	1.0523
*ν*	1.9651	2.9477	3.9303	4.9129	5.8954
*β*_*t*_	0.4271	0.5606	0.5596	0.5246	0.4810
100*δ*	24.39	**0.7780**	0.9497	7.15	14.87

Bold entry indicates the *t* most consistent with the observed data on *α*,*β* and *λ*

**Table 5:** *μ*=5,*ν*=15,*t*=8,*λ* ^−1^=0.532632,*α*=0.53869147,*β*=0.03107084

par ∖time *t*	4	5	6	7	8	9	10
*μ*	6.2529	5.4956	5.2256	5.1247	5.1051	5.1313	5.1861
*ν*	7.5099	9.3873	11.2648	13.1423	15.0198	16.8972	18.7747
*β*_*t*_	0.0281	0.0317	0.0324	0.0318	0.0306	0.0292	0.0277
100*δ*	9.43	2.18	4.22	2.33	**1.39**	6.00	10.97

Bold entry indicates the *t* most consistent with the observed data on *α*,*β* and *λ*

It can be seen, at least in these diverse examples, that the inference procedure generally identifies the correct value of *t*, and good estimates of *μ* and *ν*.

## Discussion

The introduction of sweeps consisting of alternating jumps and deletions, with time-invariant parameters *ν* and *μ*, provide us with an improved possibility of solving the fractionation model completely. We do announce such a solution, though it has much room for improvement. Though the exponential distribution of visible segment lengths should be easy to establish analytically, it is also possible that the gamma distribution of invisible segment lengths could be proved, including the *α* and *β* parameters as a function of the number of sweeps. Depending on the functional form of such a solution, the inference of *t*,*μ* and *ν* might be amenable through closed form formulae rather than the quadratic modeling. Nevertheless, we have succeeded for the first time in inferring the parameters of a fractionation model, albeit a “one-sided” model and a continuous analog of more realistic discrete fractionation models.

## Conclusions

Aside from theoretical improvements, the first priority for this work should be the return to a discrete gene-order model of fractionation with the insights gained in the current report. This should be extended to, or at least tested on simulations of, two-sided fractionation models with subgenome dominance (higher deletion rates on one copy of the genome than the other).
